# Space-Based Sentinels for Measurement of Infrared Cooling in the Thermosphere for Space Weather Nowcasting and Forecasting

**DOI:** 10.1002/2017SW001757

**Published:** 2018-04

**Authors:** Martin G. Mlynczak, Delores J. Knipp, Linda A. Hunt, John Gaebler, Tomoko Matsuo, Liam M. Kilcommons, Cindy L. Young

**Affiliations:** 1 NASA Langley Research Center, Hampton, VA, USA; 2 Aerospace Engineering Sciences Department, University of Colorado Boulder, Boulder, CO, USA; 3 SSAI, Hampton, VA, USA

## Abstract

Infrared radiative cooling by nitric oxide (NO) and carbon dioxide (CO_2_) modulates the thermosphere’s density and thermal response to geomagnetic storms. Satellite tracking and collision avoidance planning require accurate density forecasts during these events. Over the past several years, failed density forecasts have been tied to the onset of rapid and significant cooling due to production of NO and its associated radiative cooling via emission of infrared radiation at 5.3 μm. These results have been diagnosed, after the fact, through analyses of measurements of infrared cooling made by the Sounding of the Atmosphere using Broadband Emission Radiometry instrument now in orbit over 16 years on the National Aeronautics and Space Administration Thermosphere, Ionosphere, Mesosphere Energetics and Dynamics satellite. Radiative cooling rates for NO and CO_2_ have been further shown to be directly correlated with composition and exospheric temperature changes during geomagnetic storms. These results strongly suggest that a network of smallsats observing the infrared radiative cooling of the thermosphere could serve as space weather sentinels. These sentinels would observe and provide radiative cooling rate data in real time to generate nowcasts of density and aerodynamic drag on space vehicles. Currently, radiative cooling is not directly considered in operational space weather forecast models. In addition, recent research has shown that different geomagnetic storm types generate substantially different infrared radiative response, and hence, substantially different thermospheric density response. The ability to identify these storms, and to measure and predict the Earth’s response to them, should enable substantial improvement in thermospheric density forecasts.

## Plain Language Summary

This paper describes a new class of observations that could dramatically improve the forecasts of conditions in space during “space weather” events. A network of orbiting sensors would detect the infrared heat radiation from Earth’s atmosphere that is generated when energy from the Sun carried by particles in the solar wind interacts with Earth’s high atmosphere (above 100 km high). These sensors would serve as an early warming system, relaying their observations to ground stations. These observations would then be fed into forecast models for space weather, just as regular weather satellites track storms near Earth’s surface. The new observations would improve the ability to track space objects such as the International Space Station, all orbiting satellites, and orbital debris during the disturbed space weather conditions caused by storms from the Sun.

## 1. Introduction

An outstanding problem in solar-terrestrial physics is the accurate, real-time prediction of neutral density and temperature changes in the thermosphere during geomagnetic storm events. These predictions are needed for accurate forecasting of aerodynamic drag on space vehicles and orbital debris, so as to enable operational space agencies to keep track of these objects during times when density, and hence aerodynamic drag, change dramatically. The problem may seem simple at first; energy input during the geomagnetic storm heats the thermosphere and causes it to expand, increasing density at fixed altitude. Heat conduction and infrared radiation remove the energy and should allow the thermosphere to return to its original state in a matter of days, depending on storm intensity. However, the processes are substantially more complicated, and as stated in the 2013 Heliophysics Decadal Survey, Solar and Space Physics: A Science for a Technical Society (National Academy of Sciences, 2013), “theory still cannot explain how the global thermosphere ‘inflates’ several hours after the onset of high-latitude heating, nor have studies yet captured the subsequent cooling with the fidelity that is needed to predict changes in satellite orbits occurring during magnetic storms.” Furthermore, “overcooling” events (e.g., Lei et al., 2012) result in the collapse of density, rather than its enhancement, shortly after storm onset. The resolution of this problem is identified as a Key Science Goal of the Decadal Survey. In 2016, National Aeronautics and Space Administration (NASA) established a “virtual institute” team of experts under its Living with a Star program to address challenges associated with real-time forecasts (“nowcasts”) of thermospheric density during geomagnetic storms. NASA virtual institutes (Guhathakurta, 2017) are designed to bring together teams of researchers to solve problems that are too big and complex for an individual researcher to solve. The thermospheric density virtual institute team was charged with examining the state of the art and making recommendations for substantial improvements. This paper is one of the products of the virtual institute activity.

The concept to be developed in this paper is that infrared radiation emitted by nitric oxide (NO) and carbon dioxide (CO_2_) in Earth’s thermosphere is a very sensitive indicator of changes in temperature and density, via the basic physics responsible for the generation of infrared radiation. Based on studies of the past 16 years of NO and CO_2_ cooling rate data from the Sounding of the Atmosphere using Broadband Emission Radiometry (SABER) instrument on the NASA Thermosphere, Ionosphere, Mesosphere Energetics and Dynamics (TIMED) satellite, it is quite clear now that space-based sentinels monitoring the infrared cooling of the thermosphere would provide real-time measurements that can be used to substantially improve nowcasts of thermospheric density, temperature, and composition. These observations, coupled with the knowledge gained on cooling intensity as a function of storm type and structure, will lead to fewer failed density forecasts, and thus more accurate prediction of space vehicle positions, and fewer temporarily lost payloads and debris objects. These observations may also serve as a continued monitor of the infrared thermosphere to further the understanding of long-term solar-terrestrial coupling.

In the next section the basic physics of the generation of infrared radiation in the thermosphere is reviewed, including the “natural thermostat” concept (Maeda et al., 1989, 1992; Mlynczak et al., 2003) of NO during geomagnetic storm events. Observations of radiative cooling made by the SABER instrument during its 16-plus years (and counting, as of this writing) will be presented. Sections 3 and 4 present reviews of recent work on the relation of infrared cooling by NO and CO_2_ to failed density forecasts, thermosphere composition, temperature, and density changes and most recently to the structure of geomagnetic storms. These two sections provide evidence of the utility of real-time observations of infrared cooling to improve nowcasts during storm events. In section 5 the characteristics of the infrared measurements and instrument design considerations will be described, as well as discussions of orbit sampling needed for the proposed sentinel network, and further observing system simulation experiments that should be conducted to optimize the design and deployment strategy for the infrared sentinels. Section 6 describes potential use of the infrared cooling data in forecasting models, and section 7 is the paper summary.

## 2. Generation of Infrared Radiation in the Thermosphere

**Figure 1.**
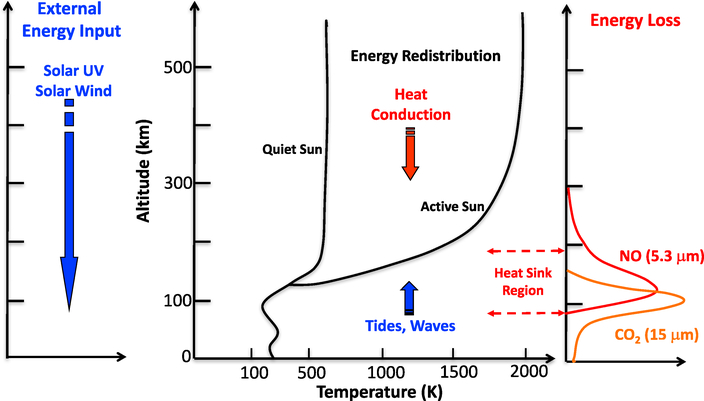
Conceptual overview of the thermosphere thermal structure and its energy budget.

Figure 1 shows an illustration of the basic processes of energy input, energy redistribution, and energy output above 100 km. The depiction of the thermal structure during times of low (quiet) and high (active) solar activity is inspired by the similar, classic figure in Banks and Kockarts (1973). Energy is input to the system externally from the Sun in the form of photons (X-rays to ultraviolet) and from particle precipitation associated with the solar wind (Figure 1, left). Energy is also input from the atmosphere below in the form of tides and waves. Molecular heat conduction removes energy from the highest altitudes down the thermal gradient to regions where the atmosphere is sufficiently dense for infrared radiation to occur, generally below 200 km. We refer to this region between 100 km and 200 km as the “heat sink” region as indicated in the figure, as it is this region where the vast majority of the energy is lost from the thermosphere by infrared radiation.

Emission and escape of infrared radiation is the only mechanism by which the Earth’s surface and atmosphere lose thermal energy to space. The primary infrared radiators in Earth’s thermosphere are CO_2_, NO, and atomic oxygen (O), the latter of which possesses two fine structure lines, one at 63 μm and one at 147 μm. Atomic oxygen is the dominant infrared radiator in the thermosphere above 200 km to 250 km, depending on the level of solar activity. However, at these and higher altitudes, molecular heat conduction is the dominant cooling mechanism. NO and CO_2_ emit in their vibration-rotation bands centered near 5.3 μm and 15 μm, respectively. As with all infrared radiators throughout the atmosphere, O, NO, and CO_2_ gain energy in their internal quantum states through collisions with other atmospheric species. This internal energy may be emitted spontaneously in the form of infrared photons cooling the atmosphere locally, or it may be returned back to the translational energy field in a subsequent collision, resulting in no loss of energy from the atmosphere.

In this paper, we focus on the response of NO and CO_2_ to short-term geomagnetic storm events. Mlynczak et al. (2005) showed that the infrared response of atomic oxygen 63 μm emission relative to that of NO and CO_2_ during geomagnetic storm events would be somewhat muted, because of the much weaker temperature dependence of the longer-wavelength transitions. In the thermosphere, taken here to be above 100 km, the primary mechanism by which the vibrational modes of NO and CO_2_ are excited is through collisions with atomic oxygen. P. Crutzen (Houghton, 1970) predicted this effect for CO_2_. Kockarts (1980) showed that collisions between atomic oxygen and NO followed by spontaneous emission of infrared radiation is a major cooling mechanism in the thermosphere. The basic processes for radiative cooling by NO are as follows

(1) $$\mathrm{NO}\;\left(\upsilon =0\right)+O\to \mathrm{NO}\;\left(\upsilon =1\right)+O\;\left(\Delta E=-1875\;{\mathrm{cm}}^{-1}\right)$$

(2) $$\mathrm{NO}\;\left(\upsilon =1\right)\to \mathrm{NO}\;\left(\upsilon =0\right)+h\nu \;\left(\Delta E=+1875\;{\mathrm{cm}}^{-1}\right)$$

(3) $$\mathrm{NO}\;\left(\upsilon =1\right)+O\to \mathrm{NO}\;\left(\upsilon =0\right)+O\;\left(\Delta E=+1875\;{\mathrm{cm}}^{-1}\right)$$

The rate coefficient for process ((3)), the collisional quenching rate *k*_10_, is usually what is reported in the chemical kinetics literature, in this case for NO, by Hwang et al. (2003). The rate for the reverse process *k*_01_, collisional excitation, is obtained by detailed balance, *k*_01_ = *k*_10_ exp (−Δ*E*/*k*_*b*_*T*_*K*_), where *k*_*b*_ is Boltzmann’s constant and *T*_*K*_ is the neutral kinetic temperature.

The radiative cooling rate d*Q*/d*t* is given in units of energy loss per unit volume per unit time (W/m^3^ in MKS units) and is essentially an expression from the first law of thermodynamics,

(4) $$dQ/dt=\rho \;C_{p}\;\left(dT/dt\right)=\mathrm{NO}\left(\upsilon =1\right)\times A_{10}\times E_{10}$$

where *ρ* is the density, *C*_*p*_ the heat capacity at constant pressure, and d*T*/d*t* the rate of change of neutral kinetic temperature. *A*_10_ is the Einstein coefficient for spontaneous emission of radiation and *E*_10_ is the energy of an emitted photon, for a transition from the *υ* = 1 to *υ* = 0 vibrational levels.

Solving the rate equations above for the cooling rate d*Q*/d*t*:

(5) $$\frac{dQ}{dt}=\frac{k_{10}\cdot O\cdot \mathrm{NO}\cdot A_{10}\cdot E_{10}}{A_{10}+k_{10}\cdot O}\cdot \exp\left(-\frac{2,700}{T_{K}}\right)$$

In the three rate equations and in the expression above, we are neglecting the process of radiative excitation of the NO molecule by upwelling radiation from the lower atmosphere. For NO this process is small relative to the collisional excitation rate and its neglect adds no uncertainty to the calculation of the cooling rate and very little uncertainty to the interpretation of infrared radiance measurements (such as those from the SABER instrument, discussed below). In addition, a tacit assumption in the cooling rate expression for NO is that all emitted radiation escapes the thermosphere, either to space or to the atmosphere below, where it is absorbed. Mlynczak et al. (2005) demonstrated the validity of the cool-to-space approximation.

Lastly, the formation of the NO molecule during geomagnetic storm conditions may lead to the excitation of vibrational-rotation bands of NO above the *υ* = 1 level. However, emission associated with these high-lying bands is not formally radiative cooling of the thermosphere. The infrared energy emitted originates during the chemical reactions forming NO during the storm and is a product of the conversion of chemical potential energy of the reacting species to internal quantum energy of the vibrationally excited NO molecule. As discussed in Mlynczak and Solomon (1993), such energy does not originate within or enter into the thermal (neutral kinetic energy) field. The extent to which this energy cascades down to the *υ* = 1 level may be an issue for interpretation in terms of the radiative cooling rate measurements of infrared emission from NO during an active storm time in which NO is being actively produced. These issues have been addressed in the interpretation of SABER data (Mlynczak et al., 2005). The main result is that the radiative cooling of the thermosphere by NO is accomplished by the fundamental vibration-rotation band.

Similar expressions to those above can be written for infrared cooling of the thermosphere by CO_2_, resulting in the following expression:

(6) $$\frac{dQ}{dt}=\frac{2\cdot k_{10}\cdot O\cdot {\mathrm{CO}}_{2}\cdot A_{10}\cdot E_{10}}{A_{10}+k_{10}\cdot O}\cdot \exp\left(-\frac{960}{T_{K}}\right)$$

In equation (6) the term *k*_10_ is the rate of collisional quenching of the upper vibrational state of CO_2_ due to collisions with atomic oxygen and *A*_10_ is the inverse radiative lifetime of that state. Although the symbols are the same, the values of these terms are different than for NO, as is *E*_10_, the energy of a 15 μm photon, which when expressed in Kelvin (by dividing appropriately by Boltzmann’s constant) is the term 960 in the parenthesis of the exponential. Lastly, the “2” in the numerator of equation (6) accounts for the ratio of degeneracies between the upper and lower vibrational levels of CO_2_. As CO_2_ is not created or destroyed by a geomagnetic storm, there is typically no need to consider emission from its higher lying states. In the lower thermosphere, the fundamental vibration-rotation band is essentially responsible for the entire cooling by CO_2_.

The issues regarding absorption of upwelling radiation and escape of radiation are much more serious for CO_2_, as it may not be completely in the weak-line (optically thin) limit of radiative transfer which is tacit in equation (6). The cooling rate calculations typically require complex radiative transfer modeling in which multiple layers of the atmosphere are coupled by radiative exchange under conditions of nonlocal thermodynamic equilibrium (non-LTE). Analysis of the CO_2_ cooling rates using SABER observations does account for these non-LTE effects (Mlynczak et al., 2010). A key question for interpretation of measurements of CO_2_ infrared emission from the space-based sentinels proposed in this paper is the extent to which the assumption of weak-line radiative transfer is valid above 100 km. This issue will be discussed further below.

In summary, infrared radiation during geomagnetic storm events is promptly generated in accordance with basic physical processes. As temperature increases, infrared emission from NO and CO_2_ increases nonlinearly, and much more strongly for NO than CO_2_. In addition, the substantial increase in NO abundance results in a “doubly nonlinear” increase in radiative cooling by NO. Maeda et al., (1989, 1992) first noted this in a model study. Mlynczak et al. (2003), in the first analysis of radiative cooling by NO from SABER observations, called this effect the “natural thermostat” of NO. These and subsequent observations showed that infrared energy, particularly the strongly enhanced NO emission, returns the thermosphere to near prestorm conditions in 2–3 days’ time.

## 3. Observations of Radiative Cooling From SABER

The SABER instrument was launched on the NASA TIMED satellite in December 2001 and continues to operate nominally as of this writing, over 16 years. The instrument operated over that time with an average duty cycle of 98%. SABER is a 10-channel, limb scanning, infrared radiometer (Russell et al., 1999), and the experiment is designed to observe and quantify the energy budget of the mesosphere and lower thermosphere (Mlynczak, 1996, 1997). SABER provides measurements of temperature, ozone, CO_2_, atomic oxygen, atomic hydrogen, and volume emission rates of the hydroxyl and O_2_(^1^Δ) molecules. A water vapor data product should be made public in the near future. SABER data span altitudes from the tropopause to 300 km, depending on the specific parameter.

**Figure 2.**
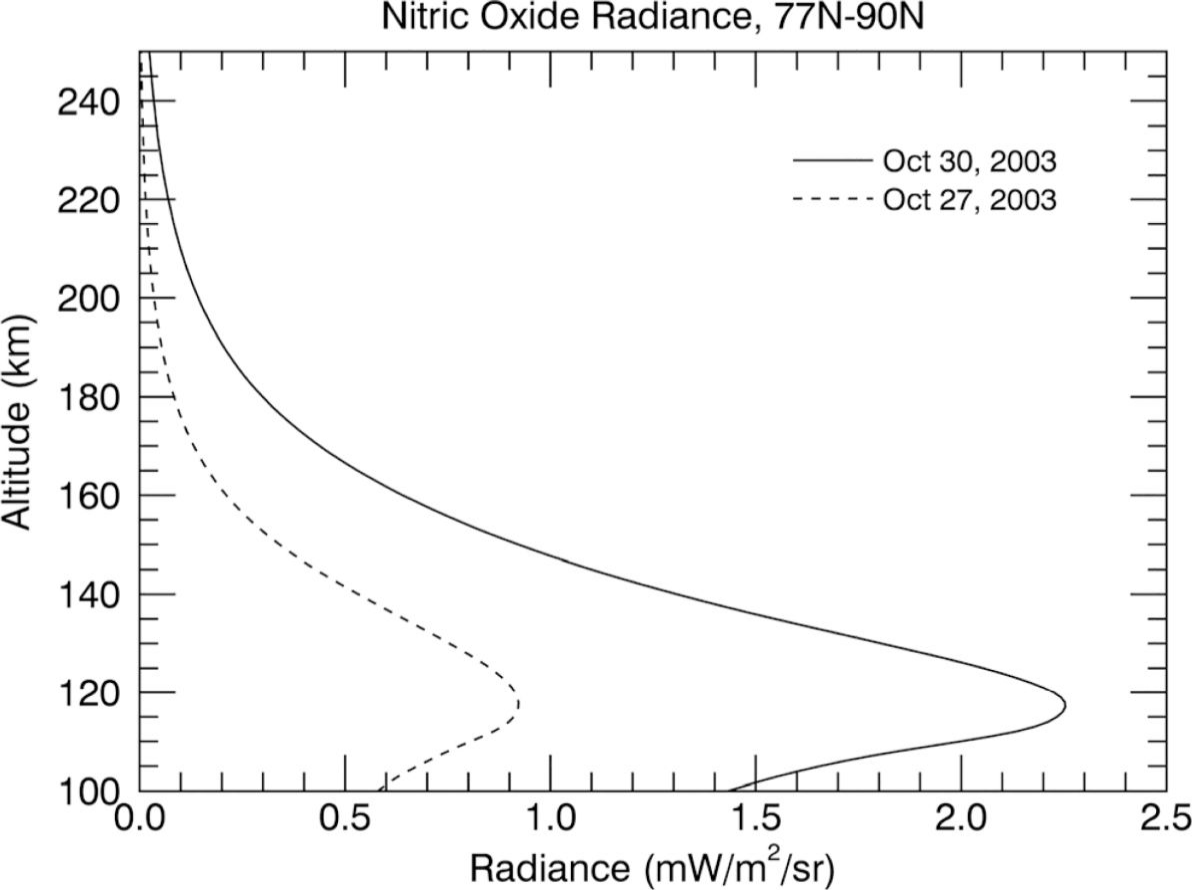
Zonal average limb radiances (mW/m^2^/sr) from nitric oxide (NO) at 5.3 μm observed by Sounding of the Atmosphere using Broadband Emission Radiometry between 77°N and 90°N during prestorm conditions (27 October 2003) and during storm conditions on 30 October 2003.

**Figure 3.**
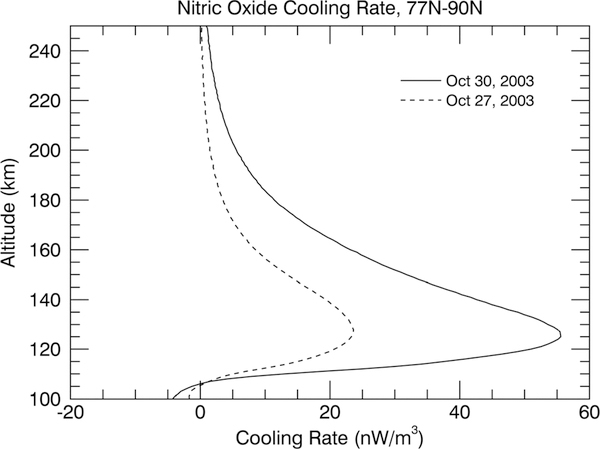
Zonal average cooling rates (nW/m3) due to nitric oxide (NO) between 77 N and 90 N observed by Sounding of the Atmosphere using Broadband Emission Radiometry during prestorm conditions (27 October 2003) and during storm conditions on 30 October 2003.

Figure 2 shows SABER infrared radiance profiles of NO during quiet and geomagnetically disturbed conditions in late October 2003, at the time of the “Halloween Storms.” The limb radiance (W m^−2^ sr^−1^) is observed to dramatically increase during the storm, indicating a large increase in the radiative cooling of the atmosphere by infrared radiation. Figure 3 shows the cooling rate of infrared radiation emitted by NO prior to and during the storm. These cooling rates (nW/m^3^) are derived (Mlynczak et al., 2005) directly from the measured radiance profiles via an Abel inversion. A factor is applied to account for the width of the spectral band-pass of the filter in the SABER NO channel. These data are averages of individual profiles recorded between latitudes of 77°N to 90°N on the indicated days. The solid curve shows the cooling rate during the storm time as compared with the dashed curve illustrating the cooling prior to the onset of the storm and illustrates the large increase in cooling due to the production of NO and increase in temperature during the storm.

**Figure 4.**
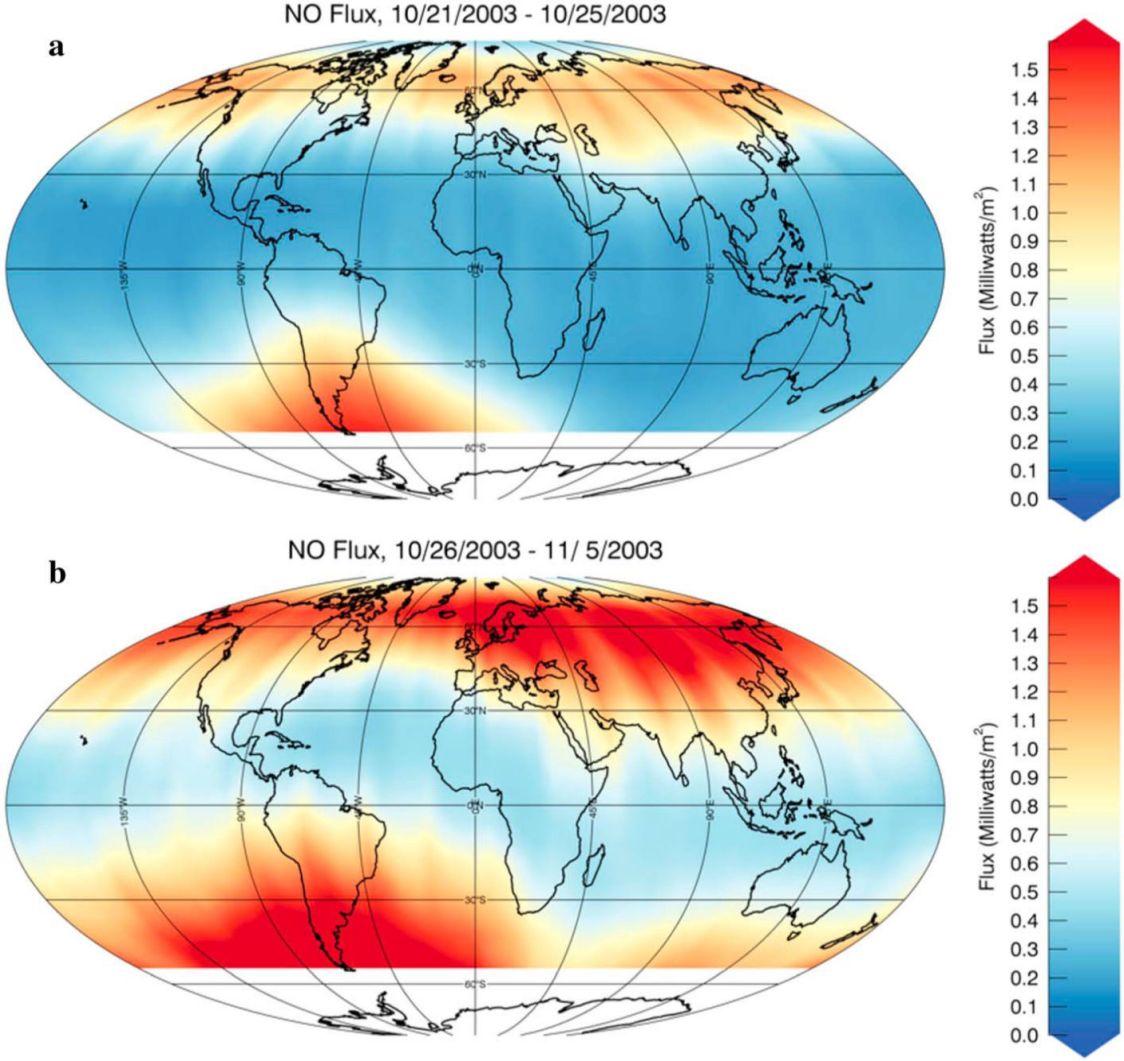
(a) Prestorm fluxes (mW/m^2^) of infrared radiation from nitric oxide (NO) observed by SABER between 21 and 25 October 2003. (b) Fluxes (mW/m^2^) of infrared radiation from NO observed by Sounding of the Atmosphere using Broadband Emission Radiometry during storm conditions from 26 October to 5 November 2003.

The increased infrared radiation due to NO is not limited to high latitudes. This is readily illustrated in Figures 4a and 4b which shows the flux (mW/m^2^) of radiation from NO that exits the thermosphere, either to the lower atmosphere or to space. Figure 4a shows the fluxes in the quiet time before the storm while Figure 4b presents fluxes during the storm. Fluxes of infrared radiation are obtained by vertical integrals of the radiative cooling rates. The range of integration is from 100 km to 140 km for CO_2_ and 100 km to 250 km for NO. Figure 4 clearly show the enhancement of NO cooling extending to middle and lower latitudes during the storm, implying that the effects of a strong geomagnetic storm are nearly global in extent. These effects in NO radiation are caused by changes in temperature and composition (NO and O), according to equations (1)–(3) above.

**Figure 5.**
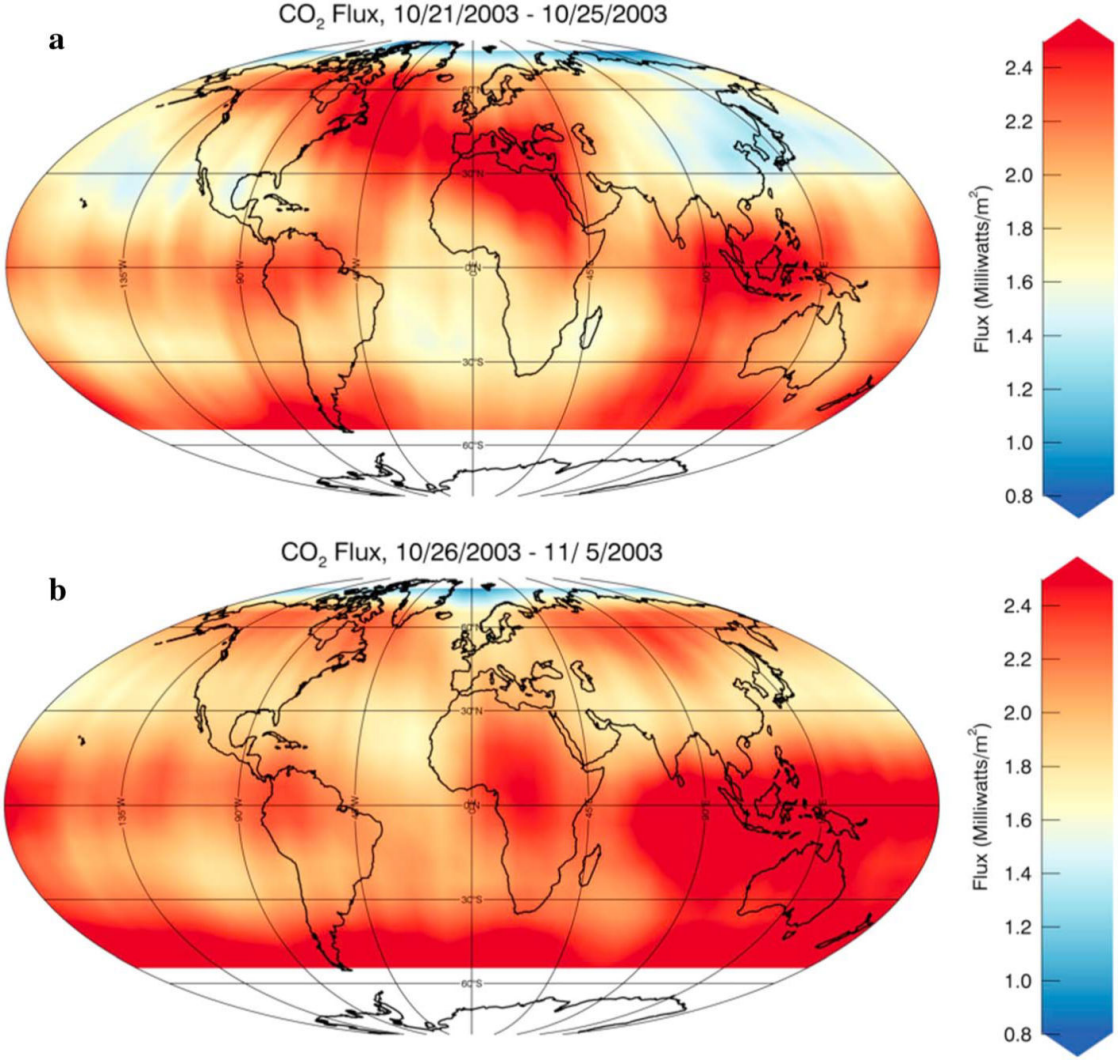
(a) Prestorm fluxes (mW/m^2^) of infrared radiation from carbon dioxide (CO_2_) observed by SABER between 21 and 25 October 2003. (b) Fluxes (mW/m^2^) of infrared radiation from CO_2_ observed by Sounding of the Atmosphere using Broadband Emission Radiometry during storm conditions from 26 October to 5 November 2003.

The infrared response is not limited to NO and also includes a significant response from CO_2_. Shown in Figures 5a and 5b are fluxes before and during the storm, respectively, for CO_2_. As with NO, the CO_2_ radiative response is evident at all latitudes, and the combined response of CO_2_ and NO determine the magnitude of the “thermostat” effect. To illustrate the relative roles of each, we show in Table 1 the total global power (TW) from eight large storms observed by SABER, and the percentage of the total due to NO and CO_2_. Specifically, Table 1 shows the radiated power above the background radiation prior to and after the storm, while Figures 4 and 5 show the total emitted radiation. For these eight cases, radiative cooling by NO is on average about 2/3 of the total in each storm. Further study of the response of CO_2_ to storm type as in Knipp et al. (2017) is essential to understanding the radiative response of the thermosphere.

## 4. Relation of Infrared Cooling to Thermospheric Density, Temperature, Composition, and Aerodynamic Drag on Space Vehicles

Initial analyses of the SABER data of radiative cooling of the thermosphere by NO and CO_2_ were focused on understanding the physics and magnitude of the cooling and the causes of its observed variations (Mlynczak et al., 2003, 2007, 2008, 2010). These observations confirmed the modeling results of Maeda et al., (1989, 1992), namely, that NO infrared cooling plays a major role in the recovery of the thermosphere subsequent to geomagnetic storm events and that the cooling also responds to both shorter and longer term solar variability. Subsequent research (Qian et al., 2010) examined the radiative cooling and density response of the thermosphere to geomagnetic forcing using numerical models, finding good agreement between observed and modeled radiative cooling by NO. Lei et al. (2011) examined the observed density response of the thermosphere during the dramatic superstorm events of October 2003 using measurements from the CHAMP (Challenging Mini-Satellite Payload) and GRACE (Gravity Recovery and Climate Experiment) satellites. They found that the observed recovery time for density was much faster than that predicted by the either the MSISE00 or TIEGCM models and was unable to explain the larger model response times. This difference was explained shortly thereafter by Lei et al. (2012) using observations of the infrared cooling by NO measured by SABER. They concluded that elevated levels of NO infrared cooling during the poststorm time period succeeded in “overcooling” the thermosphere to a colder and less dense state than existed prior to the storm. The large effects of observed NO cooling on the observed density response clearly indicate the potential for real-time observations of NO cooling to be used in “nowcasts” of aerodynamic drag on space vehicles.

The concept of using real-time observations of NO cooling for improving density forecasts was strengthened significantly by Knipp et al. (2013) who studied density forecasts for a series of “problem storms.” The density forecast in these storms was shown to fail because the forecast models did not predict the (unexpected) decreases in thermospheric density associated with production of large amounts of NO and, subsequently, the radiative cooling by NO. Forecast models typically use ground-based indices such as Ap and Dst which fail to adequately capture the interaction of the solar wind and the subsequent production of NO and its cooling of the thermosphere. Knipp et al. (2017) effectively demonstrated the challenge with predicting NO production and cooling using such indices. They showed that the strength of the NO cooling, in particular the occurrence of “overcooling,” depends directly on the properties of solar ejecta dynamics that interact with the Earth’s magnetosphere and ultimately results (or does not result) in density and cooling changes that results in “overcooling” events. Specifically, using SABER data, Knipp et al. (2017) showed that incoming coronal mass ejections led by shock and sheath structures produced large NO cooling responses. In comparison, nonshocked storms produced limited changes in density or NO cooling. Finally, Weimer et al. (2015, 2016) showed fundamental relationships between thermospheric density (atomic oxygen), the exosphere temperature, and the SABER NO and CO_2_ infrared radiative cooling. These relationships, particularly the relation between CO_2_ cooling rates and atomic oxygen variations, and the relationship between NO cooling rates and thermospheric temperatures, have their basis in the physical processes discussed in section 2. Weimer et al. (2016) specifically noted the utility of real-time measurements of infrared cooling for improvement of thermospheric density modeling.

## 5. Concept of Sentinels Measuring Infrared Cooling in Real Time for Use in Nowcasts of Thermospheric Density

The basic infrared radiation physics discussed above, followed by the real-life examples of the role infrared emission by NO and CO_2_ have in determining the density response of the thermosphere during geomagnetic storm events leads directly to the conclusion that real-time observations of the NO and CO_2_ emission could be vital new inputs to short-term thermospheric density forecasts. We will discuss first the considerations of temporal and spatial sampling, orbital characteristics, and number of infrared sensors. Then we will discuss considerations and trades that must go into the design of a small instrument for measuring the NO cooling and possibly the CO_2_ cooling as well.

### 5.1. In-Orbit Sentinel Network Concept

In this section, we report the results of an initial orbital trade study conducted to provide guidance on the required number of sentinel instruments and the characteristics of their orbits. The results of this study (and subsequent refinements), besides defining the number of instruments, will also play a role in the design of the instruments and thus play a major role in determining the overall cost of the sentinel network (including the number of launches if different orbital planes are required).

The orbital trade study begins assuming that like the SABER instrument, the sentinel sensor will need to view away from the Sun for optimal thermal stability and performance. And also, like SABER, the assumption is that each sentinel will make one complete limb view every 60 s. The trade study then involves comparison of latitude coverage, with frequent high-latitude coverage preferred, orbit altitude, and orbit inclination. Orbit altitude is limited by the current 25 year deorbit rule to less than 700 km. The best orbit from the perspective of temporal and spatial sampling turns out to be one with an inclination of 70° and 400 km altitude. This orbit is very nearly identical to the orbit of the TIMED satellite on which SABER resides, 74° inclination and 600 km altitude. The TIMED inclination was chosen to give an integral number (6) of 24 h local time samplings every calendar year. The range of latitudes observed is from 50 N to 85 S from 400 km altitude, because of the requirement to view orthogonal to the velocity vector. SABER encounters essentially the same restriction on viewing latitudes—however, the TIMED spacecraft undergoes a “yaw” maneuver every 60 days to keep SABER pointing away from the Sun. Similarly, the sentinels at 70 deg inclination would have to undergo similar maneuvers approximately every 50 days. Reaction wheel and/or gyroscope technology for small satellites to perform these maneuvers is commercially available (e.g., www.bluecanyontech.com).

For the sentinel network to be effective and have continuous viewing of both polar regions, a second orbit plane rotated 180° in RAAN (right ascension of the ascending node) from the first is required. These two orbit planes at the specified inclination and altitude, with instruments viewing away from the Sun and orthogonal to their velocity vectors, would provide pole to pole coverage. However, a single sentinel in each orbit would view a polar region approximately every 95 min. In order to have a higher cadence of polar observations, more sentinel instruments are required in each orbit plane. Three sentinels per orbit would provide polar views approximately every 30 min, while six per orbit plane (12 total) would provide views of each polar region approximately every 15 min.

The above discussion lays out several considerations for a network of sentinel instruments for observing NO cooling in real time. More detailed observation system simulation experiments are required to definitively determine the required polar revisit time for observing NO emission for improving real-time forecasts of satellite drag, and thus determining the number of sentinel instruments. Other considerations (such as the need for Sun avoidance) will be addressed in engineering trade and performance studies described below.

### 5.2. Instrument Considerations

The current SABER instrument in orbit on the TIMED satellite is a research instrument designed over 20 years ago. The instrument weighs 77 kg and consumes 75 W of power on average. It uses single element detectors (one detector for each of its 10 channels) and interference filters mounted on top of the detectors to achieve spectral isolation required for each channel. A mirror continuously scans the Earth’s limb from approximately 400 km down to 10 km below the hard surface of the Earth. Approximately 55 s are required for each individual scan during which time the spacecraft moves approximately 350 km. The mirror directs infrared radiation from the Earth’s limb into the SABER Cassegrain telescope which focuses the radiance on the detector focal plane. A profile of limb radiance is recorded in each of the 10 spectral channels during each scan. The limb radiance is sampled at a rate corresponding to every 0.4 km on the Earth’s limb, achieving a five times oversampling rate of the nominal 2 km field of view. Approximately 1,500 profiles of limb radiance are recorded in each of the 10 channels each day. The SABER instrument views the atmosphere orthogonal to the spacecraft velocity vector so that it may always be viewing away from the Sun. It does so in order to maintain a stable thermal environment in terms of its radiator temperature and to minimize thermal gradients within the instrument. Every 60 days the spacecraft rotates 180° in the yaw direction in order to keep the instrument pointed away from the Sun. The SABER radiances are calibrated with an on board blackbody. Radiometric offsets are corrected subsequent to each “space view” at 400 km altitude tangent height. The detectors are cooled to 74 K with a miniature pulse tube cryocooler. The data rate for the entire instrument (including all radiometric and housekeeping data) is a very modest 4 kB/s.

A new instrument design is required in order to achieve an affordable and sustainable infrared sentinel-for-space-weather-forecast concept. There are many trades that can be considered relative to the legacy SABER instrument in order to design a smaller, lighter, and sustainable capability for infrared radiance measurements. It is not necessary to replicate all 10 SABER channels in an infrared sentinel. In principle, from a data retrieval and scientific analysis perspective, the minimum requirement would be to measure the emission from NO between 100 km and 300 km altitude. As the emission is in the weak-line limit, the analysis of the radiance profile in terms of the vertical profile of the infrared cooling rate requires a geometric (or Abel) inversion, essentially inverting a lower-triangular matrix whose elements are the line-of-sight path lengths at each tangent altitude. The following discussion will first consider an “NO-only” sentinel, and then a sentinel capable of measuring NO and CO_2_ will be considered.

The first trade would be to examine the use of detector arrays, either one-dimensional (linear) arrays or two-dimensional arrays. This would eliminate the need for a scan mirror as the array could continuously stare at the Earth’s limb. Arrays would carry a second advantage of being able to sample the Earth’s radiance longer since each detector would effectively be measuring the same altitude during its integration time. This may reduce requirements on detector cooling, further reducing the power and heat dissipation (i.e., thermal radiators and hence mass) requirements on the sentinel instrument.

**Figure 6.**
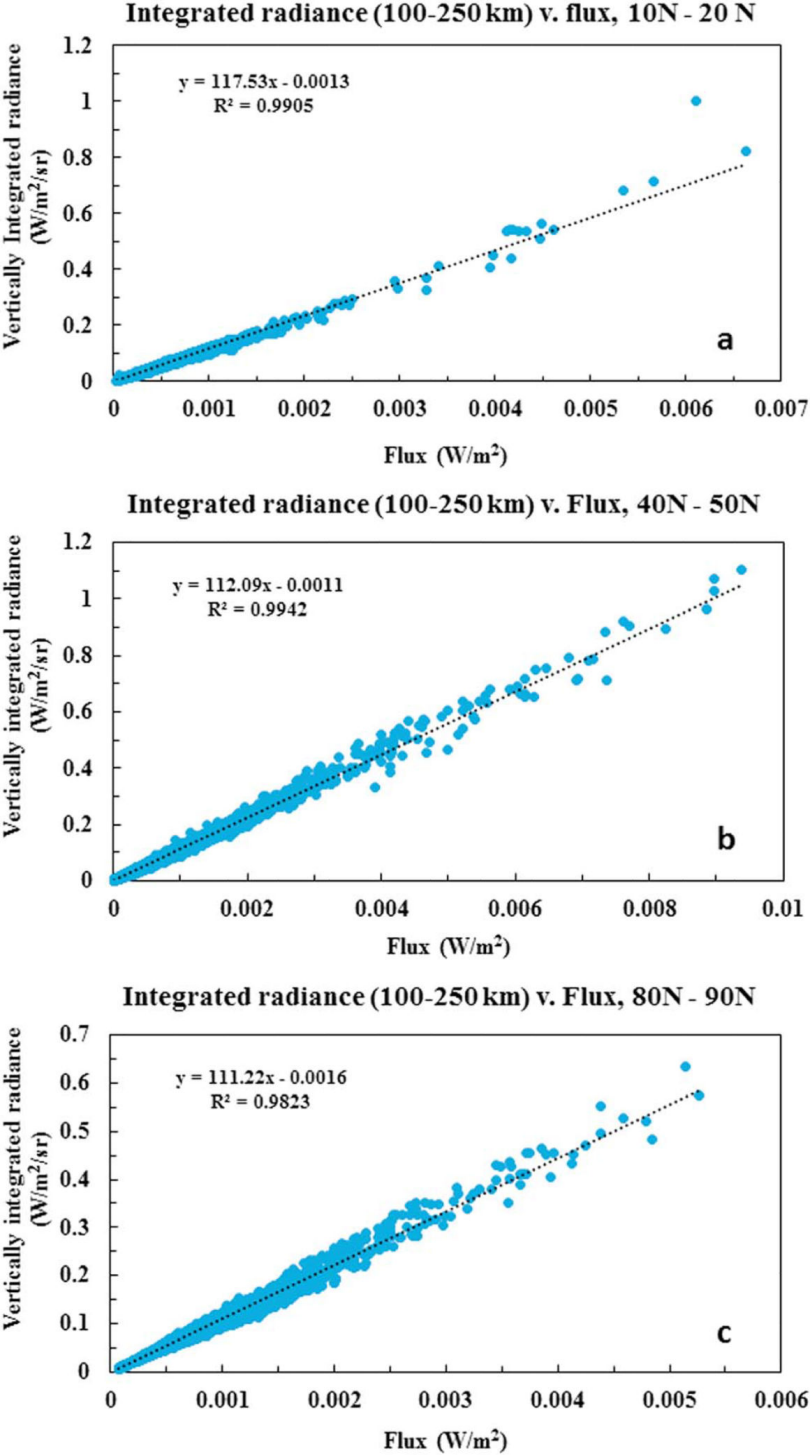
Sounding of the Atmosphere using Broadband Emission Radiometry (SABER) thermospheric NO flux (abscissa, W/m^2^) versus vertically integrated SABER limb radiance (ordinate), in three different 10° latitude bins, 10°N–20°N (a), 40°N–50°N (b), and 80°N–90°N (c). These figures imply that wide field-of-view radiances are related accurately to a geophysically significant quantity, thus allowing the infrared sentinel data to serve as both a space weather forecast tool and a monitor of solar-terrestrial coupling over the long term.

A major consideration would be the effective field-of-view size required for the instrument to perform as an infrared sentinel. SABER is designed for retrieval of vertical profiles of energy loss (W/m^3^) as the primary data product. As discussed above, the energy loss/cooling rates are vertically integrated to yield fluxes, which are then integrated meridionally to yield zonal and global power values. A sentinel instrument that viewed the entire limb from 100 km to 300 km in altitude with one or at most a few detectors would record all the NO radiance at once, further reducing cooling and sensitivity requirements on the detector. These “wide field-of-view” measurements would not be able to be inverted to vertical profiles, fluxes, or power. However, we have simulated wide field-of-view measurements by vertically integrating the high-resolution SABER limb radiances between 100 and 300 km altitude. These integrated radiances are then compared with the corresponding flux (mW/m^2^) of NO emission computed as described earlier in this paper. Figure 6 shows scatterplots of simulated wide field NO limb radiance versus flux in three different 10° latitude bins for 1 year of data, with over 40,000 radiance scans in each bin. There is nearly perfect correlation between the integrated radiance and the actual, computed flux. Thus, a wide field-of-view instrument could serve as both a storm sentinel and can provide highly accurate NO fluxes for scientific research on solar-terrestrial coupling (e.g., Knipp et al., 2017; Mlynczak et al., 2010). Given that the sentinel instruments must be low cost and sustainable over time, the field-of-view size must be a major consideration as the limb sampling rate may drive instrument mass and power to an exceptionally large degree.

A key performance metric of any infrared limb viewing instrument is its off axis or out-of-field rejection capability. SABER’s optical design is a Cassegrain telescope that focuses infrared radiation from the limb onto the detectors. SABER also has a very long baffle extending from the scan mirror to the side of the spacecraft to further limit off-axis radiation. The off-axis problem can be viewed as follows: The side of the spacecraft through which the instrument views the Earth’s limb is potentially exposed to all radiation from a 2*π* steradian hemisphere that includes the lower atmosphere and Earth’s surface. The major concern is the infrared radiance from below the tangent point. The limb radiance (see Figure 1 above) typically increases by an order of magnitude 50–100 km below the tangent point. If even 1% of the radiance from the atmosphere below the sensor falls on the detector, it could be 10% of the observed signal. Radiation from the solid earth is substantially larger and may be a significant source of out-of-field radiation. Cumulatively, the off-axis signal from the atmosphere and hard Earth below the tangent point could be comparable to, or larger than the signal of interest. New optical designs that afford compact instrumentation, minimize active and passive cooling requirements, while increasing signal-to-noise and rejecting the off-axis radiance are required.

Finally, instrument pointing accuracy and knowledge need to be determined, as does attitude stability during a limb observation. Limb scanning instruments like SABER typically require attitude stability in the axis of the limb scanning, so that spacecraft motion does not add (or subtract) apparent motion to that of the scan mirror, and thus induce pointing errors which translate to errors in registering the limb radiance profile in altitude and pressure. This is a crucial requirement for instruments like SABER in which the radiative transfer equation is mathematically inverted in order to derive temperature and minor species abundances. The nonlinear nature of the radiative transfer equation is such that small errors in pointing (and hence, pressure, and altitude) can translate into large errors in the retrieved data products. For observations of NO, which is optically thin, and for the purposes of a sentinel, pointing and attitude stability may not be a major issue, particularly if detector arrays are used instead of a scanning mirror. In addition, attitude control capabilities of small spacecraft have improved markedly in the 20 years since the TIMED satellite was designed, and thus, the pointing and stability requirements may be well within contemporary capabilities. Engineering trade studies in concert with sentinel science requirements are needed to fully assess the situation.

The initial focus of the infrared sentinel would be the measurement of emission from NO. However, as shown in Figures 5a and 5b and Table 1, CO_2_ cooling is also important in storm events. Measurement and interpretation of CO_2_ emission at 15 μm brings its own set of additional requirements which will likely add additional complexity to the development of an infrared sentinel. First, the emission from CO_2_ in the limb view, at least at tangent heights near the peak of the CO_2_ emission (100–110 km), is likely not optically thin. Thus, a simple Abel inversion like that used in the analysis of the SABER NO data will not produce correct cooling rate profiles. In addition, the non-LTE processes governing the emission are sufficiently complex that altitudes far below the peak may need to be observed so that the coupled radiative and statistical equilibrium equations may be properly solved to yield correct cooling rates. As mentioned above, this is discussed in detail in Mlynczak et al. (2010), and the SABER analyses do account for these issues. A larger scan range, and the need for nonlinear inversion techniques, will bring additional requirements on the spacecraft performance and on the data analysis algorithms.

Studies similar to those proposed for NO on vertical resolution, relation of the limb radiance to the cooling, and use of the limb radiance as a sentinel are essential to evaluate the measurement requirements to fully utilize CO_2_ as a space weather sentinel.

From an engineering and detection perspective, the lower photon energy of CO_2_ 15 μm photons (relative to NO 5.3 μm photons) may result in more stringent cooling requirements for the detector and focal plane. This may translate into larger radiator area to dissipate the heat required for the additional cooling, and of course, additional power to supply that cooling. Further science and engineering trades are required to establish the performance envelope and science return of CO_2_ infrared emission as a companion sentinel to NO.

A final consideration for a sentinel network providing real-time data is the required data rate and processing time to obtain useful data products for forecast models. As noted above, the current SABER instrument has a data rate of 4 kB/s for the entire instrument data stream. A one-channel sentinel instrument for NO would have a lower data rate. The data processing to produce a vertical profile of NO cooling rates requires solving a well-posed set of linear equations via solution of a lower-triangular matrix equation. This requires a trivial amount of computer time and may even be done on board. We therefore do not anticipate any issues with being able to provide the NO infrared cooling data in real time.

## 6. Forecasting With Real-Time Cooling Rate Data

It is important to emphasize that the real-time NO and CO_2_ cooling rate data require a forecast model to predict the expected radiative cooling rates that would be used to develop improved predictions of thermospheric density response during geomagnetic storm events. In this section, we outline an approach for developing a predictive model of infrared cooling rates based on the extant SABER data set and new, real-time observations of the NO and CO_2_ cooling rates in the thermosphere.

Neural networks are perhaps an obvious choice for their ability to model nonlinear systems such as infrared radiative cooling; however, it is difficult to physically interpret the nonlinear model found by neural networks, and quantification of uncertainty is not straightforward. Alternatively, emerging techniques from the field of system identification appear to hold significant promise for developing forecast models. In particular, Box and Jenkins (1970) and related models are often used to model engineered and natural systems. Such models use a systematic method of identifying, fitting, and checking time series models. Models of this type are explicitly formulated to use a history of system inputs and outputs to forecast system behavior.

We are interested in developing an NO forecast model that identifies the most important drivers and/or driver combinations. For a system such as the atmosphere, which has memory/inertia and responds to different inputs on different time scales, we anticipate needing a model that can capture complex dynamics. The Nonlinear AutoRegressive Moving Average with eXogenous input (NARMAX) models are a nonlinear generalization of the Box-Jenkins type models that have been used (e.g., Boynton et al., 2011, 2015) to develop mathematical models of solar wind-magnetosphere coupling and to model electron fluxes at geostationary orbit (Boynton et al., 2016). There are several aspects of NARMAX type models that differentiate them from traditional model approaches used in space physics. First, they provide a formal methodology for ranking the significance of each model driver, that is, the Orthogonal Least Squares Error Reducing Ratio algorithm, because NO flux is influenced by multiple sources and loss mechanisms such a formality is needed. Additionally, NARMAX has a formal methodology for “noise modeling.” Quantitatively, this means that the model error (*ε*) from previous steps (*ε*(*t* − 1), *ε*(*t* − 2),…) can be terms in the model equation that influence the model’s next prediction. This enables the model to more seamlessly handle uncertain or incomplete inputs, or systems that produce more unmodeled signal under different conditions. Time series models, such as NARMAX, can be developed to inform their prediction with system output history/recent variability. A NARMAX-based forecast model can be “trained” with the SABER data set and then use the observed recent time history from orbiting sentinels to produce a forecast of infrared cooling up to as much as 24 h ahead.

Finally, for forecasts of radiative cooling to be useful, thermospheric density forecast models must be able to incorporate them in near real time. Physical or empirical (e.g., index-based) relationships between infrared cooling and density must be developed for the thermospheric models to produce a more accurate density forecast. These relationships could be developed first through physics-based models simulating realistic, known storms with observed cooling response from SABER. Physical relationships between cooling and density response and storm conditions would then be quantified. From there, empirical relationships (that would naturally be computationally efficient) could be developed for operational use in conventional empirical nowcast thermospheric density models such as JB2008 (Bowman, Tobiska, Marcos, Huang, et al., 2008 and Bowman, Tobiska, Marcos, & Valladares, 2008) and forecast prediction methodologies such as *Anemomilos* (Tobiska et al., 2013). There would then be a period of testing and training the empirical models with known cooling forecast against prior failed density forecasts, specifically those already identified as having failed due to enhanced cooling by NO (e.g., Knipp et al., 2013). The end result should be improved short-term density forecast capability and forecast models that are driven by more accurate information and forecasts of the key parameter, infrared radiative cooling, that strongly controls the thermospheric density response.

## 7. Summary

Forecasting density changes in the thermosphere in response to geomagnetic storm events remains both an operational challenge and frontier of scientific research. Storm events alter the thermal structure, density, and composition of the thermosphere. In addition, observations from the SABER instrument show substantial increases in infrared radiation and infrared radiative cooling from the thermosphere associated with CO_2_ and NO during storm events. These increases are anticipated based on the fundamentals of infrared radiation physics. Multiple studies over the past decade have elucidated the importance of infrared radiation, particularly that associated with NO, in modulating the thermal and density response during geomagnetic storms. One set of studies clearly identified the lack of consideration of NO cooling as being a primary culprit in failed density forecasts. Other studies have shown clear relationships between the observed response in density and composition and the intensity of the infrared radiation from NO and CO_2_. However, infrared cooling is not directly considered as a modeled parameter in thermospheric density models used to forecast effects of geomagnetic storms. The scientific research of the past decade strongly suggests that real-time observations of NO cooling rates and CO_2_ cooling rates could offer critical information for improving real-time density and composition forecasts.

It is anticipated that thermosphere density nowcasts and forecasts will be required to become ever more accurate as time goes on. The number of spacefaring nations is growing and the amount of orbital debris is growing much faster. Operational agencies are tracking thousands of items in space, 95% of which are debris, and must often reacquire numerous objects after failed forecasts. Real-time measurements of infrared cooling by NO and CO_2_ offer the potential to significantly improve short-term density forecasts by providing a key parameter not yet considered directly in forecast models. A network of 6 to 12 smallsats would provide the necessary temporal and spatial sampling to provide real-time data for density forecasts. The challenge going forward is to design an infrared sensor capable of measuring NO and CO_2_ infrared emissions while incorporating adequate thermal control, stray light rejection, calibration accuracy, pointing accuracy, and vertical resolution. A number of science and engineering trade studies related to instrument design and measurement requirements have been identified in this paper. The anticipated data rate of an infrared sentinel and the data processing requirements, especially for NO alone, are not prohibitive from an operational, real-time perspective.

## Figures and Tables

**Table 1:** Total Global Infrared Power Radiated During Eight Major Storms Observed by Sounding of the Atmosphere Using Broadband Emission Radiometry

Year	Days	Global NO + CO_2_ power (TW)	Percentage NO	Percentage CO_2_
2003	302–304	3.03	65	35
2004	313–315	2.88	68	32
2004	207–209	2.35	63	37
2002	108–110	2.00	70	30
2015	76–80	1.74	62	38
2002	274–277	1.53	66	34
2012	67–70	0.83	66	34
2017	250–252	0.81	54	46

*Note*. Total power radiated by NO and CO_2_ and the percentage contribution to the total are indicated. NO is responsible for approximately two thirds of the total radiated power over the eight storms.

## Key Points

- Infrared radiation from nitric oxide and carbon dioxide significantly modulates thermospheric density during geomagnetic storms
- Real-time observations of infrared emission by nitric oxide and carbon dioxide will improve density forecasts
- Compact sensors will enable a network of infrared sentinels to provide data to forecast models of infrared radiation and density
